# Recombinant activated factor VIIa to treat refractory lower gastrointestinal hemorrhage in a patient with recently implanted mechanical valve: a case report

**DOI:** 10.1186/1756-0500-7-535

**Published:** 2014-08-15

**Authors:** Amr S Omar, Suraj Sudarsanan, Hesham Ewila, Ali Kindawi

**Affiliations:** Department of Cardiothoracic Surgery/Cardiac Anaesthesia & ICU Section, Heart Hospital, Hamad Medical Corporation, Doha, PO: 3050, Doha, Qatar; Department of Critical Care Medicine, Beni Suef University, Beni Suef, Egypt; Department of Anesthesia, Suez Canal University, Ismaileya, Egypt

**Keywords:** Valvular surgery, Recombinant activated factor VII, Lower gastrointestinal bleeding

## Abstract

**Background:**

Bleeding is a common complication after cardiac surgery. However, lower gastrointestinal bleeding is not usually associated with this type of surgery.

**Case presentation:**

A 50-year-old man with a history of aortic regurgitation underwent elective mechanical valve replacement under cardiopulmonary bypass. He experienced a complicated intraoperative course involving unexplained cardiac arrest following induction of anesthesia. He also developed two episodes of massive lower gastrointestinal bleeding secondary to mucosal ischemia while convalescing in the cardiothoracic surgery intensive care unit. After unsuccessful attempts to control the bleeding, exhaustion of blood products, and consideration of the high risk of mortality associated with surgery and the possibility of early- and long-term surgical complications, the decision was made to administer two successive doses of recombinant activated factor VII at 60 mcg/kg. Hemostasis was achieved without adverse systemic or valvular effects.

**Conclusions:**

A favorable outcome was achieved after administration of recombinant activated factor VII, which controlled the patient’s severe lower gastrointestinal bleeding. This outcome suggests the need to raise awareness about the use of this drug in dire circumstances when other conventional measures fail or are unsuitable.

## Background

Postoperative bleeding is a major cause of mortality and increased length of stay in the intensive care unit (ICU). Bleeding after valvular or other complex cardiac surgeries is caused by thrombocytopenia or defects in platelet function after cardiopulmonary bypass and, rarely, disseminated intravascular coagulation and deficiencies in clotting factors. Even patients without clotting disorders who are subjected to cardiac surgery may develop difficult-to-control bleeding, especially patients undergoing re-do cardiac surgery or in whom the cardiac tissue is damaged or friable. Mediastinal bleeding after valve surgery may be controlled with administration of recombinant activated factor VII (rFVIIa) when conventional products fail [1, 2]. Lower gastrointestinal bleeding (LGIB) is anatomically defined as hemorrhage occurring distal to the ligament of Treitz [3]. Massive LGIB is usually associated with a high mortality rate. The most frequent causes of LGIB are vascular malformation, tumors, and particularly colonic diverticulosis, which may be asymptomatic or involve perforation and massive bleeding necessitating emergency colectomy [4]. Massive LGIB may be associated with high mortality, especially when occurring in elderly patients [4]. The sigmoid colon is a common site for this disease, but the pathology remains poorly understood [4]. Recombinant activated factor VII (rFVIIa) (NovoSeven; Novo Nordisk A/S, Copenhagen, Denmark) has been used in the management of LGIB [4–6]. However, there are no previous reports of its administration for acute massive LGIB in patients who have recently undergone mechanical valve implantation.

We herein report a case of massive LGIB after the completion of a complicated valvular cardiac surgery. Administration of rFVIIa controlled the bleeding. The use of rFVIIa was a desperate measure after the exhaustion of blood products and failure to control the bleeding with other drugs.

## Case presentation

A 50-year-old man was admitted for elective aortic valve replacement. The patient had severe aortic regurgitation; however, apart from hypertension, he had no other systemic diseases. The patient’s electrocardiogram showed a right bundle branch block with sinus bradycardia. Echocardiography revealed an ejection fraction of 45% to 55%, severe aortic valve regurgitation, and a dilated left ventricle. He had a normal preoperative coronary angiogram.

Immediately after routine induction of anesthesia, the patient developed intraoperative cardiac arrest. Cardiopulmonary resuscitation was started by performing emergency cardiopulmonary bypass, and the aortic valve was replaced with a mechanical valve. Postoperatively, the patient’s hemodynamic parameters were unstable and he was transferred to the ICU on inotropic support, atrioventricular sequential pacing, and an intra-aortic balloon pump. During the ICU stay, the patient developed acute kidney injury with hepatic dysfunction and deranged coagulation requiring massive transfusion of blood and blood products in the first 48Â hours postoperatively. Regular hemodialysis was initiated from the first postoperative day. By the fourth postoperative day, his renal, hepatic, and coagulation profiles had improved toward the reference ranges. An anticoagulation regimen of heparin and oral warfarin was started. Because of his depressed level of consciousness and inability to be weaned off mechanical ventilation, the patient underwent percutaneous tracheostomy on the seventh postoperative day. He gradually recovered in the ICU. On the 16th postoperative day, however, he developed fresh rectal bleeding. Urgent colonoscopy revealed multiple ischemic ulcers in the colon and a large hematoma in the right colon (FiguresÂ 1 and 2). The anticoagulants were stopped and consultation was requested with a gastrointestinal surgeon. The surgeon felt that in light of the patient’s poor general condition, extensive disease, and likely dismal outcome of pancolectomy, surgical intervention was not warranted. The patient remained stable over the next few days and was weaned from the ventilator. Upon reinstitution of anticoagulants on the 24th postoperative day, he developed massive LGIB of approximately 6Â L in the form of melena. He also exhibited hemodynamic compromise necessitating reintroduction of vasopressors and mechanical ventilation. He was transfused aggressively with 12 units of packed red blood cells, 14 units of fresh frozen plasma, 12 units of platelet concentrate, and fibrinogen. The bleeding continued unabated despite correcting the coagulopathy. A consensus was thus reached among the intensivist, hematologist, primary cardiac surgeon, and gastrointestinal surgeon to administer rFVIIa. The patient was given 60 mcg/kg of rFVIIa as a first administration after failure of standard treatment, which resulted in prompt cessation of bleeding. He remained stable for the next 48Â hours. On the 26th postoperative day, he developed another bout of melena (approximately 2Â L). After the coagulation parameters had been corrected, a similar dose of rFVIIa was administered. This stopped the bleeding, and the patient gradually recovered. His anticoagulation treatment regimen was resumed after 1Â week, and he had no further episodes of bleeding. His prosthetic valve function was monitored with regular transthoracic echocardiography throughout these periods of coagulopathy. However, his ICU course was further prolonged by supervening sepsis. He was treated with colistin for *Acinetobacter baumannii* identified in the urine and with meropenem for *Enterobacter cloacae* in the sputum; he gradually improved upon prompt administration of these antimicrobials. The patient was finally transferred out of the ICU on the 45th postoperative day after full functional recovery and was discharged from the hospital after another week of convalescence in the surgical step-down unit. Since discharge, he has been followed regularly in the warfarin clinic for adjustment of his anticoagulant dose. Repeat colonoscopy after 3Â months revealed healing ulcers with no evidence of further bleeding episodes. The patient’s laboratory values before and after administration of rFVIIa are noted in TableÂ 1.

**Figure 1:**
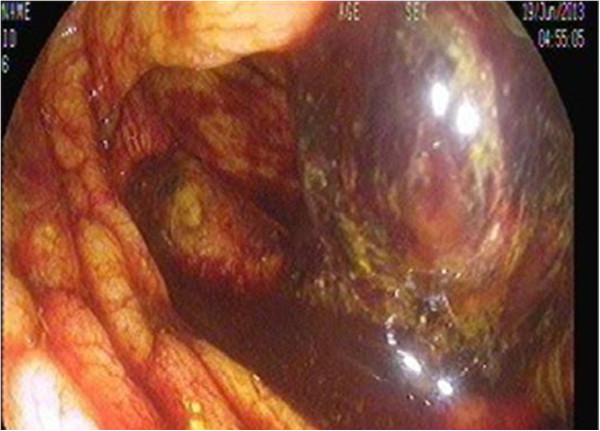
**Sigmoidoscopy showing a large hematoma in the cecum.**

**Figure 2:**
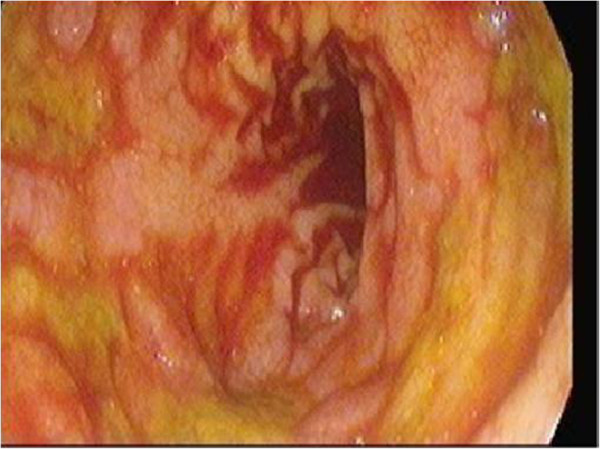
**Sigmoidoscopy showing multiple ulcerations in the ascending colon.**

**Table 1:** **Patient laboratory value before and after administration of rFVIIa**

	On admission	Before 1 ^st^bleeding episode	24 hours after VIIa	Before 2 ^nd^ bleeding episode	24 hours after VIIa
Creatinine	96	763	639	605	474
Hb	14.8	7.6	10.1	10.9	9.9
Hematocrite	44.5	20.9	29.5	32.1	31.1
Platelets	190	90	104	132	164
WBCs	8	7.2	8.7	9.6	10.9
RBCs	5.4	2.5	3.4	3.6	3.2
PTT	34.4	32.8	30.1	30.9	32.7
INR	1.1	1.6	1.1	1.2	1.1
Fibronegen		1.7	2.74	3.31	3.04
D-Dimer		4.31	2.53	2.64	2.83
Na	137	138	145	144	138
K	4.5	4.9	3.7	4.1	4
Ca ++	2.1	2.18	2.41	2.37	3.35

Approved by the Institutional Research Ethics Committee of Hamad Medical Corporation State of Qatar (protocol #13347/13).

## Discussion

The drug rFVIIa was developed to treat patients with congenital hemophilia or inhibition of factor VIII or IX. Multiple individual case reports have described the successful use of rFVIIa as a hemostatic drug in the setting of uncontrolled bleeding, even without preexisting coagulopathy [7–9]. Its mechanism of action involves enhancing hemostasis at the site of injury where tissue factor is produced without systemic activation of the coagulation cascade. At pharmacologic levels, rFVIIa forms a complex with tissue factor from the subendothelium and on the surface of cells at the site of tissue damage. rFVIIa also binds to the surface of activated platelets.

Factor X is activated by rFVIIa–tissue factor complex and rFVIIa on the surface of platelets, to FXa, which complexes with FVa to catalyze the conversion of prothrombin to thrombin. In addition, rFVIIa has been used to treat diverse congenital and acquired hemostatic disorders with variable rates of success [4]. Its hemostatic mechanism of action is attributed either to activation of thrombin by endogenous tissue factor, which is a site-specific procoagulant action, or to activation of factor X, which binds to platelets without tissue factor, leading to thrombin formation when a large amount of rFVIIa is administered. Thrombin activation leads to fibrin stabilization and fibrin degradation inhibition [7]. The success of rFVIIa as a curative therapy has been demonstrated in many case reports in which hemostasis was achieved in patients with life-threatening LGIB [5], and it has been used successfully to control mediastinal bleeding after cardiac surgery [7, 8].

The use of cardiopulmonary bypass in valve replacement and coronary artery bypass graft surgery may be complicated by excessive bleeding [9]. This cannot explain the bleeding in our patient because he did not exhibit significant mediastinal bleeding and because his LGIB occurred late in the course. However, ischemia, age, and atherosclerosis may explain such an event [4].

While rFVIIa has been reported to control life-threatening colonic bleeding, to our knowledge it has never been used in a patient with a recently implanted mechanical cardiac prosthetic valve, which confers additional risk. The potential advantage of rFVIIa in patients with LGIB is that it offsets the high-risk surgery undertaken to control bleeding; up to 43% of patients have no identifiable bleeding point [10, 11]. Although a standard dose of rFVIIa has not been identified, doses of 40, 60, and 90 mcg/kg have been given in various situations and have produced different outcomes in terms of reduction of bleeding, decreased use of blood products, and enhanced surgical intervention results [11, 12]. In light of the limited data on optimal dosing, we were advised to initiate a moderate dose in the present case and repeat it if necessary. Some practitioners have advocated repeated administration after 6 hours for clot stabilization [5, 12], but this was not needed in our patient. Because of the high cost of rFVIIa, a pragmatic approach is required in using this drug; the cost of intervention must be weighed against both the relative risk and burden on the blood bank. Complications associated with the use of rFVIIa include thrombosis, which had an incidence as high as 13.8% in one report [13], and increased mortality. The risk of thrombosis was higher in our patient because of the existing mechanical valve. Postoperative obstruction and thrombosis of the valve are not uncommon, occurring at rates of 0.3% to 1.3% and as high as 10% in nonobstructive thrombosis, respectively [14]. Mechanical valves are usually associated with a lower rate of thrombosis than are bioprosthetic valves, but still carry a risk. This risk can be reduced with appropriate anticoagulation [15]. Because surgical intervention in the present critically ill patient carried a high risk of mortality, we used rFVIIa as a rescue measure when routinely used blood products failed to stop the bleeding and alternatives were limited. The relatively high cost of rFVIIa mandates its proper use; notably, it is licensed to be used in patients with hemophilia, and its use outside this spectrum is considered to be off-label [12]. It has been argued that the hemostatic effectiveness of rFVIIa as a therapeutic intervention remains unproven and that clinical trials should guide licensing for indications other than hemophilia [16]. However, the safety of rFVIIa was investigated in a study by von Heymann et al. [8] in which no thromboembolic complications were encountered after administration of this drug to patients in the study group. This result led the authors to acknowledge the safety of last-resort therapy with rFVIIa, but not to support its routine use when conventional therapy is available [8]. Moreover, a local procoagulant effect of rFVIIa without activation of the systemic coagulation cascade has been mentioned in several studies [17, 18]. These studies found that rFVIIa may provide effective hemostasis by acting locally at the site of vessel damage, where it combines with tissue factor to generate thrombin.

## Conclusion

A favorable outcome was achieved after the administration of rFVIIa to control severe LGIB after running out of options in a clinically deteriorating patient. This point to the need to raise awareness about the use of this drug as a last resort desperate measure to control bleeding which persists despite conventional management.

### Key messages

A coincidental association between massive LGIB and cardiac surgery is possible. The postoperative period of cardiac surgery may preclude surgical interventions for bleeding. rFVIIa has emerged as an effective therapeutic option in such circumstances. rFVIIa seems to be a safe option for controlling LGIB in patients with recently implanted mechanical heart valves because of its local procoagulant action. More detailed studies should be undertaken before this agent can be used routinely as first-line therapy in such instances. In the meantime, rFVIIa should be used as a last resort to control bleeding when all other options have been exhausted.

### Consent

Written informed consent was obtained from the patient for publication of this Case Report and any accompanying images. A copy of the written consent is available for review by the Editor-in-Chief of this journal.

## Abbreviations

ICU: Intensive care unit
LGIB: Lower gastrointestinal bleeding
rFVIIa: Recombinant activated factor VII.
